# Healthcare utilisation and health literacy among young adults seeking care in Sweden: findings from a cross-sectional and retrospective study with questionnaire and registry-based data

**DOI:** 10.1017/S1463423619000859

**Published:** 2019-12-10

**Authors:** Lisa Viktorsson, Pia Yngman-Uhlin, Eva Törnvall, Magnus Falk

**Affiliations:** 1 Research and Development Unit, and Department of Medical and Health Sciences, Linköping University, Linköping, Sweden; 2 Management Department, and Department of Medical and Health Sciences, Linköping University, Linköping, Sweden; 3 Primary Health Care Center Kärna, and Department of Medical and Health Sciences, Linköping University, Linköping, Sweden

**Keywords:** cross-sectional study, emergency department, healthcare utilisation, health literacy, primary healthcare, registry study, Sweden, young adults

## Abstract

**Aim:**

The objective of this study was to examine young adults’ healthcare utilisation and its possible association with health literacy.

**Background:**

Many countries struggle with insufficient accessibility at emergency departments (EDs) and primary healthcare centres (PHCs). Young adults, aged 20–29 years old, account for a substantial number of unnecessary doctor visits where health literacy could be an explanatory factor.

**Method:**

This study incorporated a combined retrospective and cross-sectional study design with analysis of registry data, including all registered outpatient doctor visits between 2004 and 2014 (*n* = 1 086 432), and strategic sample questionnaire data (*n* = 207), focusing on socio-demographics, symptoms and information-seeking behaviour. Mean differences between first-year and last-year doctor visits for each age group were calculated using registry data. Fischer’s exact test was applied to questionnaire data to analyse group differences between ED and PHC visitors as well as between patients with sufficient health literacy and insufficient health literacy. Binary logistic regression was used to investigate covariation.

**Findings:**

Healthcare utilisation has increased among young adults during the past decade, however, not comparatively more than for other age groups. ED patients (*n* = 49) compared to PHC patients (*n* = 158) were more likely to seek treatment for gastrointestinal symptoms (*P* = 0.001), had shorter duration of symptoms (*P* = 0.001) and sought care more often on the recommendation of a healthcare professional (*P* = 0.001). Insufficient/problematic health literacy among young adults was associated with having lower reliance on the healthcare system (*P* = 0.03) and with a greater likelihood of seeking treatment for psychiatric symptoms (*P* = 0.002).

**Conclusion:**

Young adults do not account for the increase in healthcare utilisation during the last decade to a greater extent than other age groups. Young adults’ reliance on the healthcare system is associated with health literacy, an indicator potentially important for consideration when studying health literacy and its relationship to more effective use of healthcare services.

## Background

Many countries, including Sweden, struggle with insufficient accessibility and long wait times at emergency departments (EDs) and primary healthcare centres (PHCs) (Gillam, 2010; Sicilani and Moran, 2013). When patients are unable to secure an appointment at a PHC, seeking care at an ED may seem like the best solution even when their medical condition would not require emergency care. While years of research demonstrate the risks of denying patients’ emergency care, not denying patients’ emergency care may lead to crowding at EDs (Richardson and Hwang, 2001). ED crowding can create risks for patients by causing delays in transport and treatment, impairing access to care and affecting the patient mortality rate (Hoot and Aronsky, 2008). Reasons have been identified in both France and Hong Kong that may explain why patients seek care at EDs instead of other healthcare facilities, including PHCs. A patient may perceive his or her medical problem to be more serious than it actually is and feel that EDs offer advantages compared to PHCs. There can be difficulties getting an appointment at a PHC, there can be long distances to a PHC, and patients may perceive better prerequisites in general at EDs (Lee *et al.*, 2000; Durand *et al.*, 2012).

Health literacy is one factor shown to influence patients’ use of health services. Insufficient health literacy increases healthcare utilisation and frequency of doctor visits (Berens *et al.*, 2016; Palumbo *et al.*, 2016). Sufficient health literacy could potentially create more effective use of healthcare services and thereby lower healthcare utilisation (Mancuso, 2008; Sykes *et al.*, 2013). Health literacy is defined as the capacity to access, understand, appraise and apply different types of health information in order to make decisions concerning healthcare, disease prevention and health promotion in everyday life (Sørensen *et al.*, 2012). Sufficient health literacy is crucial for individuals’ empowerment and has a key role among health determinants (Dahlgren, 1991; WHO, 2016). Overall, health literacy can be divided into three subgroups: (1) An individual’s ability to read health information and health instructions, that is, functional health literacy; (2) An individual’s ability to comprehend and assimilate information, that is, interactive health literacy; or (3) An individual’s ability to critically analyse information, that is, critical health literacy (Nutbeam, 2008). This study focuses on interactive and critical health literacy, since functional health literacy is assumed to be a lesser problem due to Sweden’s nine years of compulsory schooling.

Several studies examining different types of healthcare settings indicate that patients’ doctor visits are inappropriate or unnecessary, as stated in a systematic review by Carret *et al* (Carret *et al.*, 2009). But what visits are deemed inappropriate? Depending on criteria for defining inappropriate healthcare use – which are numerous – the prevalence varies from 10 to 90% (Carret *et al.*, 2009). Multiple studies exploring unnecessary healthcare use indicate young adults – defined as being 20– 29 years of age – account for a substantial number of unnecessary and non-urgent doctor visits. Young adults also tend to be less satisfied with the healthcare services they receive (Hammond *et al.*, 2004; Carret *et al.*, 2009; Fortuna *et al.*, 2010; Davey *et al.*, 2013). Simultaneously, research questions the accuracy of defining ED visits as unnecessary by illustrating poor identification of non-urgent patients (Durand *et al.*, 2011; Honigman *et al.*, 2013). Patients aged 20–29 years old not only represent many of the defined unnecessary healthcare visits but also face new obstacles as part of the transition from adolescence to adulthood, which entails a multitude of new social and economic responsibilities and behaviours, including independent healthcare utilisation (Irwin, 2010; Neinstein and Irwin, 2013). This transition period means that young adults are in fact a vulnerable group and thus present an age-related category of interest when analysing healthcare utilisation.

Based on the findings in foregoing research that indicate inaccuracy in defining healthcare visits as unnecessary, this study aims to examine young adults’ healthcare utilisation as a whole. On the basis of healthcare utilisation being defined as, *the quantification or description of the use of services by persons for the purpose of preventing and curing health problems, promoting maintenance of health and well-being, or obtaining information about one’s health status and prognosis* (Carrasquillo, 2013), the aim was to examine both quantification and description of healthcare utilisation: quantification in terms of number of doctor visits over time and description in terms of exploring healthcare-seeking patterns with regard to doctor visits.

### Aim

To examine young adults’ healthcare utilisation, the study sought to explore if young adults’ proportion of doctor visits have increased over time compared to other age groups and what characterises young adults’ patterns of healthcare-seeking behaviour (prior to a random doctor visit). Further, to examine whether health literacy is associated with young adults’ healthcare-seeking patterns.

## Methods

### Design

**Figure 1.**
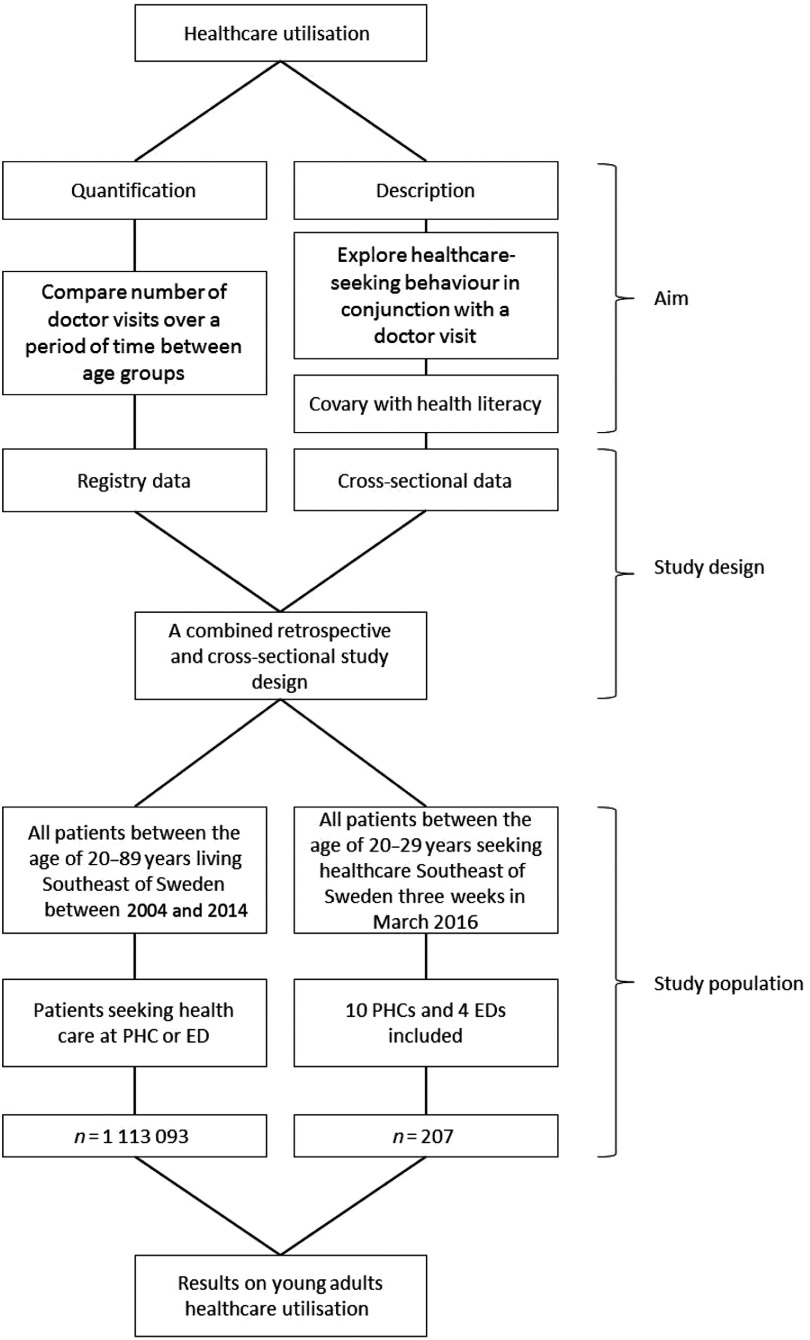
Flow chart on study design

The study employs a combined retrospective and cross-sectional study design with analysis of population and questionnaire-based data (Figure 1).

### Setting

In relation to other European countries, primary care represents a relatively small proportion of total healthcare in Sweden (Vårdanalys, 2017). However, primary care, together with emergency care, is the main entries into Swedish healthcare. Primary care is divided into three parts: PHCs, outpatient specialist services and telephone- or web-based healthcare services. The system is structured so that the majority of patients can seek healthcare at PHCs, except in cases of urgent symptoms or injury when EDs are appropriate. In Sweden, healthcare is publicly funded and the healthcare organisation is divided into 21 county councils distributed across the country. Three county councils in Southeast Sweden, Kalmar, Jönköping and Östergötland with a combined population of just over one million citizens were included in this study.

### Study population and data collection

#### Registry data

Anonymised population-based registry data comprised of all registered outpatient healthcare doctor visits were collected from each county council. For data collection, the Care Data Warehouse, an administrative healthcare registry, was used in Östergötland (Wiréhn *et al.*, 2007). In Kalmar and Jönköping, anonymised extracts from the patient record system, Computer Software Management and Information Center (COSMIC Intelligence), were used. Data collected included outpatient healthcare doctor visits, date of visit, sex, age, healthcare unit and diagnosis. Years studied were 2004–2014 in Östergötland, 2011–2014 in Kalmar and 2012–2014 in Jönköping; the shorter time frame of study in the latter two counties was due to their previous use of another form of computerised patient records.

#### Questionnaire data

The questionnaire survey was carried out during March 2016 in 14 healthcare units: 4 EDs and 10 PHCs. This selection of healthcare units was intended in order to attain variation in geographic location and number of patients listed.

Inclusion criteria were as follows: patients between 20 and 29 years of age, making a doctor visit for a non-emergent problem (according to healthcare professionals) and being able to speak and understand Swedish. Study participants included patients who visited EDs or PHCs during a period of three weeks, who fulfilled the inclusion criteria and who agreed to participate when asked. Of 496 questionnaires distributed to eligible patients, 207 were returned, yielding a response rate of 42%.

Questionnaires were placed at the reception area in each healthcare unit and distributed to patients who matched the inclusion criteria. Participants also received an informational letter and were given the opportunity to either answer the questions on-site and return the questionnaire directly to the reception desk or answer elsewhere and send it back in a pre-paid envelope.

The research team developed a modified, shortened questionnaire based on Backman’s interview study of non-urgent patients (Backman *et al.*, 2008) for the study. The questionnaire contained 20 questions regarding socio-demographics, symptoms and information-seeking behaviour. The questionnaire included five multiple-choice questions regarding reasons for visiting healthcare, self-care management and types of information sources used before visiting, and four questions with a four-grade-scale response option, for example, not anxious at all to very anxious and not bothered at all to very bothered. To secure the questionnaire’s validity, a pilot test was carried out using 10 individuals between 20 and 29 years of age. Each of the 10 individuals first answered the questionnaire separately before participating in a group discussion about the questionnaire’s comprehensibility, clarity and extent, whereupon a few adjustments were made. Besides the 20 questions modified from Backman’s interview study, the questionnaire also included the 16-item Swedish Health Literacy Survey (HLS-EU-Q16 SE).

HLS-EU-Q16 SE is a validated Swedish 16-item short version questionnaire of the European health literacy questionnaire (HLS-EU-Q47) used to measure health literacy (Sørensen *et al.*, 2012). The HLS-EU-Q47 is based on questions from three health domains: healthcare, disease prevention and health promotion and four dimensions of competences related to health: the ability to access, understand, appraise and apply health information. The shortened Swedish version covers those 3 domains and 4 dimensions with 16 questions comprised of 4 response options: very easy, fairly easy, fairly difficult and very difficult.

To register the outcome of participants’ doctor visits, consent was requested to review their medical record for diagnosis. In Sweden, the International Classification of Diseases and Related Health Problems-Tenth Revision (ICD-10) is used for diagnosis classification.

### Statistical analysis

#### Registry data

Descriptive analyses were conducted for registry data to calculate means of number of doctor visits with standard deviations and standard errors. Mean differences were calculated between first-year doctor visits and last-year doctor visits for each age group in every county council for both PHCs and EDs. Confidence intervals at 95% for mean differences were calculated.

#### Questionnaire data

All variables in the questionnaire were dichotomised, and Fischer’s exact test was used to analyse socio-demographics, symptoms and information-related group differences among ED and PHC visitors and patients with sufficient health literacy versus those with insufficient and/or problematic health literacy. Binary logistic regression was used to assess how certain variables covariate and may predict the outcomes of having insufficient and/or problematic health literacy and seeking healthcare at EDs. Outcomes were tested in a hierarchical model. The first step was to analyse crude rates in a simple logistic regression. The second step was to conduct a backward stepwise multiple logistic regression. Variables with less than five in one group were left out. A double-sided *P*-value of *P* < 0.05 was considered statistically significant. Data were analysed using SPSS version 25.0 software (Chicago, IL, USA).

When analysing the HLS-EU-Q16 SE, index calculation was done in three steps, as suggested by Wangdahl *et al.* (Wangdahl *et al.*, 2014). First, the response categories were dichotomised so that ‘very easy’ and ‘fairly easy’ were merged and given the value of 1; the responses ‘very difficult’ and ‘fairly difficult’ were merged and given the value of 0. The responses ‘don’t know’ were treated as missing. Second, a sum score of the response values was calculated. The highest scores, 13–16, were put in the category ‘sufficient critical health literacy’, the second highest scores, 9–12, were put in the category ‘problematic critical health literacy’ and the lowest scores, 0–8, were given the category ‘insufficient critical health literacy’. These three categories were later dichotomised to ‘sufficient health literacy’, 13–16 scores and ‘insufficient/problematic health literacy’, 0–12 scores because of too few participants with low scores.

## Results

### Registry data

A total of 8 664 320 doctor visits and 1 086 432 unique patients were identified in the databases for the years studied. Of the total doctor visits, 56% were made by women and 44% were made by men. The results presented a significant increase of doctor visits over time in almost every age group in both EDs and PHCs for Östergötland and Kalmar, though not in Jönköping where only a single increase was found (Table 1). In all county councils, the mean value of number of doctor visits increased in the 20–29 age group, for both EDs and PHCs: Östergötland increased from 0.77 to 0.92, Kalmar from 1.02 to 1.23 and Jönköping from 1.17 to 1.22. According to ICD-10, the most common main diagnosis for 20–29 year olds at EDs was R10, abdominal and pelvic pain. From 2004 to 2014, this diagnosis increased from 8 to 14.2%. In 2014, 13.8% of 20–29 year olds at EDs were diagnosed R104X, other and unspecified abdominal pain not otherwise specified, with 63% of these patients being females. The R104X diagnosis decreased with increasing age, dropping slightly to 12.5% for the 30–39 age group and falling all the way to 4% for the 80–89 age group. The second most common diagnosis in 2014 was R074, chest pain unspecified, with 3.1%. Among PHC patients, the main diagnoses were more scattered. The most common diagnosis was J06, acute upper respiratory infection, with 3.6%.

**Table 1.** Doctor visits at emergency departments and primary healthcare centres compared over time and between age groups. Note the various time intervals

Healthcare setting	County council	Age group	Year		Difference	
			Mean		Mean difference	CI
Emergency departments	Östergötland		2004	2014		
		20–29	0.07	0.19	0.12	0.10–0.14
		30–39	0.06	0.18	0.11	0.09–0.13
		40–49	0.06	0.18	0.11	0.09–0.13
		50–59	0.07	0.20	0.13	0.11–0.15
		60–69	0.09	0.24	0.15	0.13–0.17
		70–79	0.14	0.36	0.23	0.20–0.25^a^
		80–89	0.23	0.64	0.41	0.37–0.45^a^
	Kalmar		2011	2014		
		20–29	0.16	0.24	0.08	0.05–0.11
		30–39	0.14	0.20	0.06	0.04–0.09
		40–49	0.14	0.20	0.06	0.04–0.09
		50–59	0.15	0.22	0.07	0.04–0.10
		60–69	0.20	0.28	0.08	0.05–0.11
		70–79	0.31	0.43	0.12	0.08–0.16
		80–89	0.53	0.69	0.16	0.12–0.21^a^
	Jönköping		2012	2014		
		20–29	0.20	0.21	0.01	−0.01 to 0.04
		30–39	0.16	0.18	0.02	−0.01 to 0.04
		40–49	0.16	0.17	0.01	−0.02 to 0.03
		50–59	0.19	0.19	<0.01	−0.02 to 0.03
		60–69	0.23	0.24	<0.01	−0.02 to 0.03
		70–79	0.36	0.35	−0.01	−0.04 to 0.02
		80–89	0.61	0.64	0.02	−0.02 to 0.06
Primary healthcare centres	Östergötland		2004	2014		
		20–29	0.71	0.73	0.03	0.01–0.05
		30–39	0.86	0.91	0.05	0.03–0.08
		40–49	0.87	0.93	0.06	0.04–0.09
		50–59	1.02	1.06	0.04	0.02–0.07
		60–69	1.22	1.26	0.04	0.02–0.07
		70–79	1.68	1.60	−0.08	−0.12 to −0.05^a^
		80–89	2.07	2.11	0.03	−0.01 to 0.08
	Kalmar		2011	2014		
		20–29	0.86	0.99	0.13	0.09–0.16
		30–39	1.01	1.12	0.11	0.07–0.15
		40–49	1.06	1.21	0.14	0.11–0.18
		50–59	1.23	1.42	0.18	0.15–0.22
		60–69	1.47	1.66	0.19	0.16–0.22
		70–79	1.84	2.02	0.19	0.15–0.23
		80–89	2.08	2.28	0.21	0.15–0.27
	Jönköping		2012	2014		
		20–29	0.97	1.01	0.04	0.02–0.07
		30–39	1.11	1.12	0.01	−0.02 to 0.04
		40–49	1.17	1.20	0.03	0.00–0.06
		50–59	1.36	1.36	<0.01	−0.03 to 0.03
		60–69	1.56	1.55	−0.01	−0.04 to 0.02
		70–79	1.91	1.89	−0.02	−0.06 to 0.01
		80–89	2.34	2.42	0.09	0.04–0.14

a Statistical significance (<0.05) compared to reference group 20–29 year olds

### Questionnaire data

In total, 207 patients completed the questionnaire. Characteristics of the participants are described in Table 2. Of the 207 participants, 50 were excluded for the health literacy analysis due to more than 2 missing data points on the HLS-EU-Q16 SE, including 43 ‘don’t know’ responses. Consent to obtain patients’ medical records in order to register the outcome of the doctor visit (diagnosis) was given by 106 patients. Of these 106 patients, the most common group of diagnoses (at 32%) were ‘Symptoms, signs and abnormal clinical and laboratory findings, not elsewhere classified’, R00-R99, with half of those patients diagnosed with abdominal and pelvic pain unspecified (R10.4 and R10.4X). Injury or poisoning was the second largest group of diagnoses (15%), followed by respiratory system (9%), musculoskeletal system (8%) and mental and behavioural disorders (7%).

**Table 2.** Characteristics of the study population for questionnaire-based data

Variables	Questionnaire in total, *n* = 207			HLS-EU-Q16 SE, *n* = 157		
	*n*	(%)	Dropouts	*n*	(%)	Dropouts
Sex						
Male	80	39	–	58	37	–
Female	127	61		99	63	
Age						
20–24	121	59	4	93	61	4
25–29	82	40		60	39	
Education						
Primary school	14	7	6	10	6	2
Secondary school	143	71		111	72	
University/College	44	22		33	22	
Other	1	1		1	1	
Housing						
Marriage/Cohabitation	96	48	8	76	50	4
One-person household	46	23		35	23	
Living with parent(s)	55	28		41	27	
Other	2	1		1	1	
Country of origin						
Sweden	182	88	–	137	87	–
Other	25	12		20		
HLS-EU-Q16 SE Score (mean: 13, SD: 2.7)						
0–8				9		
9–12				49		
13–16				99		

There were several differences between patients seeking healthcare at EDs compared to PHCs (Table 3). Gastrointestinal symptoms were most common at EDs, while respiratory and psychiatric symptoms were more frequent at PHCs. Almost 50% of ED patients had symptoms for less than 24 h compared with only 15% at PHCs. ED patients received a health professional’s recommendation to seek help prior to the ED visit more often than PHC patients did.

**Table 3.** Comparing patients seeking healthcare in emergency departments with patients seeking healthcare in primary healthcare centres using Fisher’s exact test

Variables (N)	Total *n* (%)	ED *n* (%) 49 (24)	PHC *n* (%) 158 (76)	*P*-value < 0.05
Sex (207)				
Male	80 (39)	18 (37)	62 (39)	0.867
Country of birth (207)				
Sweden	182 (88)	44 (90)	138 (87)	0.804
Age (207)				
20–24	121 (60)	30 (61)	91 (59)	0.868
Education (201)				
>Secondary school	44 (22)	12 (26)	32 (21)	0.546
Employment (207)				
Working	135 (65)	30 (61)	105 (67)	0.498
Unemployed	9 (4)	1 (2)	8 (5)	0.689
Student	68 (33)	22 (45)	46 (29)	0.055
Sick leave	16 (8)	1 (2)	15 (10)	0.125
Parental leave	7 (3)	1 (2)	6 (4)	1
Type of symptom^a^ (207)				
Respiratory	32 (15)	3 (6)	29 (18)	**0.042**
Circulatory	8 (4)	4 (8)	4 (3)	0.092
Muscle and skeletal	31 (15)	9 (18)	22 (14)	0.494
Trauma	18 (9)	5 (10)	13 (8)	0.772
Gastrointestinal	39 (19)	19 (40)	20 (13)	**<0.001**
Skin	18 (9)	3 (6)	15 (10)	0.573
Genital and urinary tract	10 (5)	2 (4)	8 (5)	1
Psychiatric	25 (12)	0	25 (16)	**<0.001**
Other	45 (22)	8 (16)	37 (23)	0.329
Attempt self-care^a^ (207)				
Resting	124 (60)	36 (74)	88 (56)	**0.030**
Stayed home from work/school	81 (39)	20 (41)	61 (39)	0.867
Medication	87 (42)	24 (49)	63 (40)	0.320
Physical activity	38 (18)	9 (18)	29 (18)	1
Supporting bandage	14 (7)	3 (6)	11 (7)	1
Naturopathic drug	29 (14)	5 (10)	24 (15)	0.484
Diet	33 (16)	6 (12)	27 (17)	0.508
Tried nothing	35 (17)	6 (12)	29 (18)	0.388
Symptoms, health and healthcare				
⪯24 h (197)	45 (22)	22 (48)	23 (15)	**<0.001**
Inconvenience (198)	164 (79)	39 (87)	125 (85)	1
Anxious about symptoms (203)	85 (41)	23 (48)	62 (40)	0.403
Anxious about health in general (200)	26 (13)	3 (6)	23 (16)	0.097
Healthcare within last two months (204)	65 (31)	16 (33)	49 (32)	1
Trust in healthcare (203)	56 (27)	13 (28)	43 (28)	1
Information and advice				
Healthcare professional’s recommendation to seek care (205)	66 (32)	30 (61)	36 (23)	**<0.001**
Medical advisory service as source of information (184)	166 (80)	43 (96)	123 (89)	0.249
Health literacy				
Insufficient/problematic health literacy (157)	58 (28)	10 (26)	48 (40)	0.128
Diagnosis^b^				
R00-R99 (ICD-10 symptom diagnosis) (106)	34 (16)	13 (54)	21 (26)	**0.013**

a Answer for more than one alternative possible (sum of individuals is based on the total number of people who answered one or more alternative).

b Only 106 patients gave approval to their medical records, that is, diagnosis.

A third (37%) of all patients answering the HLS-EU-Q16 SE had insufficient and/or problematic health literacy. This group had less trauma symptoms and more psychiatric symptoms than did patients with sufficient health literacy. Having insufficient and/or problematic health literacy was also associated with being more anxious about health in general and having less trust in healthcare (Table 4).

**Table 4.** Comparing patients having sufficient health literacy with patients having insufficient/problematic health literacy using Fisher’s exact test

Variables (N)	Total *n* (%)	Sufficient health literacy *n* (%)	Insufficient/problematic health literacy *n* (%)	*P*-value < 0.05
Sex (157)				
Male	58 (37)	36 (36)	22 (38)	0.865
Origin (157)				
Sweden	137 (87)	89 (90)	48 (83)	0.220
Age (157)				
20–24	93 (61)	55 (57)	38 (68)	0.229
Education (154)				
>Secondary school	33 (21)	22 (23)	11 (19)	0.686
Employment (157)				
Working	93 (59)	57 (58)	36 (62)	0.617
Unemployed	7 (5)	7 (7)	0	**0.047**
Student	56 (36)	33 (33)	23 (40)	0.491
Sick leave	12 (8)	3 (3)	9 (16)	**0.009**
Parental leave	7 (5)	6 (6)	1 (2)	0.261
Type of symptom (157)				
Respiratory	28 (18)	18 (18)	10 (17)	1
Circulatory	7 (5)	5 (5)	2 (3)	1
Muscle and skeletal	22 (14)	15 (15)	7 (12)	0.642
Trauma	16 (10)	14 (14)	2 (3)	**0.032**
Gastrointestinal	30 (19)	17 (17)	13 (23)	0.405
Skin	13 (8)	10 (10)	3 (5)	0.375
Genital and urinary tract	8 (5)	6 (6)	2 (3)	0.711
Psychiatric	18 (12)	5 (5)	13 (22)	**0.002**
Other	31 (20)	15 (15)	16 (27)	0.065
Attempt self-care^a^ (157)				
Resting	98 (62)	65 (66)	33 (57)	0.308
Stayed home from work/school	60 (38)	34 (34)	26 (45)	0.234
Medication	70 (45)	44 (44)	26 (45)	1
Physical activity	29 (19)	15 (15)	14 (24)	0.202
Supporting bandage	13 (8)	9 (9)	4 (7)	0.769
Naturopathic drug	24 (15)	18 (18)	6 (10)	0.252
Diet	29 (19)	16 (16)	13 (22)	0.395
Tried nothing	24 (15)	12 (12)	12 (21)	0.172
Symptoms, health and healthcare				
⪯24 h (150)	32 (21)	21 (22)	11 (20)	0.838
Inconvenience (147)	126 (86)	77 (82)	49 (93)	0.091
Anxious about symptoms (155)	69 (45)	42 (43)	27 (47)	0.618
Anxious about health in general (154)	25 (16)	11 (11)	14 (25)	**0.039**
Healthcare within last two months (157)	51 (33)	29 (29)	22 (38)	0.292
Trust in healthcare (155)	46 (30)	35 (36)	11 (19)	**0.029**
Information and advice				
Healthcare professional’s recommendation to seek care (156)	53 (34)	35 (35)	18 (32)	0.726
Medical advisory service as source of information (141)	129 (92)	83 (93)	46 (86)	0.359
Seeking care				
Primary healthcare centres	119 (76)	71 (72)	48 (83)	0.128
Diagnosis				
R00-R99 (ICD-10 symptom diagnosis) (85)	26 (31)^b^	15 (28)	11 (34)	0.630

a Answer for more than one alternative possible (sum of individuals is based on the total number of people who answered one or more alternative).

b Only 106 patients (24 at ED and 82 at PHC) gave approval to their medical records, that is, diagnosis.

When put into a multivariate model, trust in healthcare and anxiety about health in general was shown to covariate with seeking care for psychiatric symptoms. A multivariate model for ED patients showed that each of the variables’ gastrointestinal symptoms having symptoms for less than 24 h and receiving a healthcare professional’s recommendation to seek care interacted with being an ED patient (Table 5).

**Table 5.** Odds ratios (OR) for having insufficient/problematic health literacy and for being an emergency department patient, respectively

	Emergency department patient, *n* = 207					Insufficient/problematic health literacy, *n* = 157				
		Crude		Final model			Crude		Final model	
Variables	*n*^a^	OR	95% CI	OR	95% CI	*n*^a^	OR	95% CI	OR	95% CI
Background										
Sex										
Man	80	1		1		58	1			
Women	127	1.11	0.57–2.16	0.20	0.04–0.92*	99	0.94	0.48–1.83		
Age										
20–24	121	1				93	1			
25–29	86	0.92	0.47–1.77			64	0.62	0.31–1.24		
Origin Sweden										
No	25	1				20	1			
Yes	182	1.28	0.45–3.60			137	0.54	0.21–1.39		
>Secondary school										
No	163	1				124	1			
Yes	44	1.30	0.61–2.80			33	0.79	0.35–1.77		
Worker										
No	72	1				64	1			
Yes	135	0.80	0.41–1.55			93	1.21	0.62–2.34		
Student										
No	139	1				101	1			
Yes	68	1.98	1.03–3.84*			56	1.31	0.67–2.57		
Symptoms										
Respiratory										
No						129	1			
Yes						28	0.94	0.40–2.20		
Muscle Skeletal										
No	175	1				135	1			
Yes	31	1.38	0.59–3.24			22	0.77	0.29–2.01		
Trauma										
No	148	1								
Yes	18	1.26	0.43–3.73							
Gastrointestinal										
No	167	1		1		127	1			
Yes	39	4.52	2.15–9.52*	5.10	1.14–22.76*	30	1.43	0.63–3.20	2.76	1.08–7.04*
Psychiatric										
No						139	1			
Yes						18	5.43	1.82–16.17*	7.80	2.01–30.32*
⪯24 h										
No	152	1		1		125	1			
Yes	45	5.10	2.46–10.58*	11.53	2.95–45.10*	32	0.88	0.39–2.00		
Prior to visit										
Tried resting										
No	83	1								
Yes	124	2.20	1.09–4.47*							
Healthcare professional’s recommendation to seek care										
No	139	1		1						
Yes	66	5.26	2.65–10.44*	13.25	3.11–56.45*					
Healthcare in general										
Trust										
No	147	1				111	1			
Yes	56	1.01	0.49–2.08			46	0.42	0.19–0.90	0.34	0.13–0.87*
Health in general										
Anxious										
No						132	1			
Yes						25	2.64	1.10–6.30*		
Diagnose										
R00-R99										
No	72	1								
Yes	34	3.43	1.34–8.82*							

* Significant.

a Numbers may not reflect the total, due to participants dropping out.

## Discussion

The present study sought to examine young adults’ healthcare utilisation and patterns of healthcare-seeking behaviour. The results show that healthcare utilisation over time, in terms of doctor visits, has increased among young adults at both PHCs and EDs in Southeast Sweden. However, the increase among young adults was not significantly higher than any other age group. Healthcare utilisation among young adults differed between ED and PHC patients in terms of seeking treatment for gastrointestinal and psychiatric symptoms, duration of symptoms and seeking care on the prior recommendation of a healthcare professional. Insufficient and/or problematic health literacy among young adults was associated with having less trust in healthcare and a greater prevalence of seeking help for psychiatric symptoms.

This study does not support the theory that young adults utilise healthcare more than any other age group, at least in terms of doctor visits. Moreover, most patients visited EDs only after receiving a recommendation from a healthcare professional and not solely of their own volition. While it is common that patients display care-seeking behaviour caused by a perceived serious condition, there is also a considerable number of patients who seek care at EDs only after being referred by their general practitioner (Land and Meredith, 2013). Furthermore, referrals from medical advisory services to EDs are shown to be less likely for patients in the 20–39 age group, due to their non-urgent classification. Nurses working with telephone healthcare often act as gatekeepers trying to counteract overcrowding at EDs and PHCs (Hakimnia *et al.*, 2014; Cook *et al.*, 2015). The relationship between being an ED patient and seeking care after prior counselling with a healthcare professional could indicate that young adults are not needlessly seeking care at EDs in disproportionate numbers. On the contrary, this actually suggests that young adult ED patients seek ED care quite appropriately.

More than half of the patients visiting EDs had symptoms for more than 24 h. This could be a potential indication of inappropriate ED use. However, this may also be true for other age groups and not solely a characteristic of young adults’ behaviour. That is an inquiry not addressed in this study but may warrant future research. According to The National Board of Health and Welfare in Sweden, emergency healthcare is defined as acute injury or illness that requires action within hours, or at the most 24 h after the initial occurrence (Socialstyrelsen, 2013). Perhaps, whether or not conditions are considered acute is insufficiently communicated to patients; what may be considered obvious to healthcare professionals may not be as obvious to patients.

Gastrointestinal symptoms were more likely to be the primary complaint for ED patients than for PHC patients, and abdominal pain was also the most frequent cause of seeking healthcare at EDs. Nearly one-fifth of the 106 patients who gave consent to screen for diagnosis received a diagnosis of unspecified abdominal and pelvic pain. These findings, together with the high prevalence of unspecified abdominal pain diagnoses, imply a possible need for further investigation of young adults and symptoms of abdominal pain. When diagnosed with non-specific abdominal pain, research has shown that re-evaluation is clinically relevant, with a change of diagnosis occurring in just over one-fifth of patients (Boendermaker *et al.*, 2018). Gastrointestinal symptoms could potentially be a sign of gynaecological problems like endometriosis, which is greatly underdiagnosed (Ng and Fraser, 2013). These symptoms could also be a sign of ulterior or later developed psychiatric problems, both of which warrant further examination (Bohman *et al.*, 2010; Koloski *et al.*, 2016).

More than a third of patients included for health literacy analysis had insufficient and/or problematic health literacy. This number is comparable to Sørensen *et al.*’s findings from the European health literacy survey (HLS-EU-Q47) in which 48% of the total sample had insufficient and/or problematic health literacy, indicating a good consistency. Sørensen *et al.*’s (2015) study covered eight countries with results ranging between 29% in the Netherlands and 62% in Bulgaria. Findings from this study also showed insufficient and/or problematic health literacy was related to patients seeking care for psychiatric and gastrointestinal symptoms. Earlier research studied the connection between mental illness and poor health among adults with addiction, confirming a weak yet important relationship between potential mental illness and poor health literacy (Lincoln *et al.*, 2006). Further studies are required to examine how health literacy impacts mental illness, or vice versa, and what might be their possible effects. Considering that patients with insufficient and/or problematic health literacy also have lower trust in healthcare, and it is of interest and importance to further investigate young adults’ level of health literacy and the possible reasons accounting for it. Research has shown that health literate healthcare organisations are crucial to improve health outcomes (Palumbo, 2016). The present study showed no connection between insufficient health literacy and whether patients sought help at EDs, unlike other studies (van der Heide *et al.*, 2015; Palumbo *et al.*, 2016). This could be due to different healthcare contexts, for instance, Sweden, in contrast to many other countries, has a well-developed telephone and web-based healthcare service that delivers thorough, easy-to-understand information on symptoms as well as advice for self-care (Souza-Junior *et al.*, 2016). This service could be responsible for reducing the proportion of low health literacy patients seeking care at EDs. Notably, it is unclear whether previous studies apply to critical health literacy or just functional health literacy. To this point, the existing research is limited in regard to health literacy in Sweden. More research within this area could enable comparisons of health literacy between healthcare-seeking patients and the general population.

The strength of this study is primarily in the extent of the catchment area, which includes a good distribution of healthcare units participating in the cross-sectional part of the study, both in terms of size and location, combined with the registry data that constitute a total survey of doctor visits at EDs and PHCs in the studied region.

The study has some important limitations. A larger study population selection for the questionnaire could have enabled more subgroup analysis and may have revealed further differences between having and lacking adequate health literacy. This study aimed at reaching 400 participants but was unable to do so due to difficulties registering participants and a high number of dropouts. Despite a relatively low number of participants, several differences were identified between the subgroups. Moreover, despite the large amount of dropouts, representativeness can be argued for in the questionnaire data, since registry data in terms of diagnosis match well with the diagnosis results from the questionnaire. Another limitation was the inability to get data for all the years requested for the registry study from all three county councils, since Jönköping and Kalmar switched to new electronic medical record systems in the middle of study period. The fact that the county council of Jönköping was the exception in terms of increased doctor visits over time at EDs was probably due to the short time period studied. Finally, treating ‘don’t know’ answers as missing data points when measuring health literacy may be considered dubious, since relatively many respondents were removed from the analysis due to too many missing points. However, when using only a 16-point scale, including respondents who answered several questions with ‘don’t know’ could bias the results because of uncertainty, if answering ‘don’t know’ is a sign of having inadequate health literacy.

## Conclusion

Healthcare utilisation, in terms of number of doctor visits, among young adults has increased during the last decades, but not significantly more than in other age groups. Symptom durations of more than 24 h could potentially be an indication of inappropriate use of ED healthcare; further research is needed on the subject. Investigating how insufficient health literacy affects patients’ trust in healthcare could be helpful in fostering more positive attitudes among patients towards healthcare settings and lead to more effective use of healthcare services.
